# Nurse‑led horticultural activities as an early intervention for mild‑to‑moderate depressive symptoms among youth in Hong Kong: an exploratory randomised controlled trial

**DOI:** 10.1186/s12889-026-27376-3

**Published:** 2026-04-14

**Authors:** Po Yan Sin, William Ho Cheung Li, Hong Chen, Xinyi Xu

**Affiliations:** The Nethersole School of Nursing, Faculty of Medicine, the Chinese University of Hong Kong, Hong Kong SAR, China

**Keywords:** Community Mental Health Services, Psychiatric Nursing, Adolescent, Depressive Symptoms, Anxiety, Self esteem, Quality of Life

## Abstract

**Background:**

Studies have revealed an increasing prevalence of depressive symptoms among youth globally, including in Hong Kong, posing a serious threat to their mental health and quality of life. While it has been shown that horticultural activities are promising for reducing depressive symptoms in adults and older populations, their effectiveness among youth—particularly in Chinese communities—remains underexplored. This study examined the feasibility, applicability and effectiveness of horticultural activities in reducing depressive symptoms among Hong Kong youth, as well as reducing anxiety and enhancing self-esteem and quality of life.

**Methods:**

The intervention group attended four weekly 2-h nurse-led structured horticultural sessions on a local farm. The control group received regular mental health parameters monitoring by the researcher and a mental health information pamphlet. Outcomes, including depressive and anxiety symptoms, self-esteem and quality of life, were measured at baseline (T_0_), post-intervention (T_1_) and 4 weeks later (T_2_). The feasibility of the intervention was assessed through recruitment metrics and participants’ feedback.

**Results:**

Between October and December 2023, 108 Hong Kong Chinese youth aged 15–24 years who self-reported depressive symptoms, were equally and randomly assigned to either the intervention (*n* = 54) or control group (*n* = 54). The intervention was feasible and well accepted, with an eligibility rate of 94.2%, a recruitment rate of 77.7%, and retention rates of 88.9% at T_1_ and 69.4% at T_2_. Horticultural activities significantly reduced the severity of depressive symptoms (*β* = −1.207, 95% CI: −1.820 to − 0.043, *P* = .028), with the most notable improvement observed between T_0_ and T_1_ (*P* = .05, generalised estimating equation [GEE] analysis).

**Conclusions:**

Horticultural activities are a feasible, low-cost and acceptable intervention for reducing depressive symptoms among Hong Kong youth. Given horticultural activities’ potential to promote mental well-being, their integration into community-based mental health programmes should be considered. Further large-scale trials are recommended to validate their broader psychological benefits.

**Trial registration:**

Chinese Clinical Trial Registry ChiCTR2300075575 (registered on 8 September 2023).

**Supplementary Information:**

The online version contains supplementary material available at 10.1186/s12889-026-27376-3.

## Background

### Depressive symptoms among youth

Over the last two decades, the global incidence and prevalence of depressive disorders in youth (i.e. those aged 15 to 24 years) have nearly increased by 50% [1]. This disorder has also contributed to a steady increase in disability-adjusted life years (DALYs), reflecting a growing loss of years of healthy life among youth as a result of depression [1].

This situation is particularly concerning in Hong Kong, where an estimated 51.9% of secondary school students [2] and 40% of university students report experiencing depressive symptoms [3]. It has been asserted that the susceptibility to depressive symptoms among these age groups stems from both endogenous and exogenous factors. Endogenously, hormonal changes during puberty—such as fluctuations in testosterone, cortisol and oestradiol—can cause emotional instability [4]. The rapid development of the limbic system also elevates emotional sensitivity, increasing the risk of mental health issues [5].

In terms of exogenous factors, academic and social pressures, which are particularly intense in the highly competitive society of Hong Kong, further exacerbate this risk. For example, the heavy workloads in school and university and high parental expectations regarding scholastic achievement in Hong Kong have been strongly linked to depressive symptoms [6, 7]. These demands often result in limited time being available for rest and leisure. Additionally, navigating new relationships and romantic experiences, which are key parts of the lives of young people, can contribute to psychological distress.

### Mental healthcare system in hong kong and its limitations

To address the mental health of youth in Hong Kong, the Hong Kong government has implemented a three-tiered mental healthcare system [8]. First, at the universal level, schools offer workshops and activities to promote mental well-being for all pupils. Second, at the selective level, targeted support is provided to at-risk students through counsellors and social workers. Third, at the indicated level, clinical care is provided to those with more severe needs via psychiatrists and psychologists.

However, the first of these, namely, the universal services, are rather limited. Specifically, many school-based initiatives rely on passive, one-off activities—such as reading posters or writing thank-you cards—that lack engagement and personal reflection [9]. Studies have shown that these approaches often fail to meaningfully impact students’ mental health.

To enhance the effectiveness of current measures applied in Hong Kong’s schools and universities to prevent the development of depressive symptoms, this study proposes integrating horticultural activities as an innovative, engaging alternative.

### Horticultural activities

Horticultural activities involve taking care of plants and related tasks such as harvesting, crop art and food preparation. These activities have been implemented, predominantly in adults and the elderly, in countries such as South Korea [10], the United States [11], Japan [12] and Italy [13], with promising results in reducing depressive symptoms. A recent meta-analysis found a moderate effect size (standardised mean difference [SMD] = − 0.67) of horticultural activities for reducing depressive symptoms in adults [14], suggesting their potential for broader application.

However, there has been limited research on whether horticultural activities could exert an ameliorative impact on depressive symptoms in youth. Of the 30 studies reviewed on the benefits of horticultural activities for mental health included in a meta-analysis [14], only two focused on youth [15, 16], while one additional study [17] explored the effects of horticultural activities on adolescents with anorexia nervosa. These studies, conducted in Italy, South Korea and Iran, involved small samples and reported inconsistent effect sizes (SMD range: −0.92 to 0.56). Thus, the effectiveness of horticultural interventions for youth remains unclear.

There has also been little research on the mental health benefits of horticultural activities in Hong Kong. Those trials that have been performed in Hong Kong have focused on adults [18] and the elderly [19, 20], leaving a knowledge gap regarding the benefits for youth.

Horticultural activities offer a range of advantages for ameliorating depressive symptoms. Unlike traditional therapies such as cognitive behavioural therapy and interpersonal therapy—which may carry a stigma—horticultural programmes can be framed as recreational or extracurricular, reducing the risk of labelling [21]. They are also resource-efficient, group-based and promote social interaction, communication and collaboration—factors known to support mental well-being.

Importantly, horticultural activities align with the WHO’s Comprehensive Mental Health Action Plan 2013–2030 [22], which advocates community-based, non-clinical interventions. By offering an accessible and engaging approach, horticultural activities may help prevent the progression of depressive symptoms into clinical depression among youth.

### Theoretical framework

The Attention Restoration Theory (ART) [23, 24] has been widely applied in nature-based interventions to reduce negative emotions, including among youth [25, 26]. Kaplan identified four stages of attention: directed attention, effortless attention, directed attention fatigue and restored attention. Prolonged use of directed attention—common during stress or cognitively demanding tasks—leads to fatigue, impairing focus and cognitive function. Recovery requires exposure to restorative environments that promote effortless attention, allowing the replenishment of mental resources.

Attention fatigue is closely linked to depressive symptoms [27]. Youth frequently engage in directed attention due to academic, social and extracurricular pressures. Sustained cognitive load can impair emotional regulation and executive function, increasing vulnerability to self-blame, hopelessness and social withdrawal—core features of depression.

Horticultural activities align with this framework by offering immersive, restorative environments that foster soft fascination—gently engaging attention without effort. By removing youth from daily stressors, these activities support the transition from attention fatigue to restored attention, promoting emotional recovery and psychological well-being.

Although relatively few studies have explicitly applied ART to the management of depressive symptoms among youth, this framework is particularly well suited to this population. ART provides a non-clinical, nature-based approach to alleviating psychological distress without formally positioning participants as recipients of psychiatric treatment. This characteristic is especially relevant for youth, who may be reluctant to engage in conventional mental health services because of concerns about stigma, identity formation, or peer perceptions.

The low degree of perceived stigmatisation associated with ART-based interventions is of particular importance within Chinese cultural contexts, where mental illness continues to be socially sensitive and where being labelled as “mentally unwell” may discourage help-seeking. By framing horticultural activities as recreational, educational, or lifestyle-oriented experiences, ART allows psychological restoration to occur in a culturally acceptable and developmentally appropriate manner.

Furthermore, depressive symptoms among youth often present along a subclinical or transient continuum and do not necessarily progress to formally diagnosed depressive disorders. In this context, ART may be more appropriate than therapy-oriented frameworks—such as the biopsychosocial or cognitive–behavioural models—that are traditionally designed for clinical treatment. ART emphasizes prevention, emotional regulation, attentional recovery, and psychological resilience, which align closely with the early-intervention needs of youth experiencing mild to moderate depressive symptoms. As such, ART offers a theoretically sound foundation for community-based, preventive mental health strategies targeting young populations.

## Methods

### Aims and objectives

This exploratory randomised controlled trial (RCT) was intended to evaluate the applicability and feasibility of a standardised horticultural activities programme for youth in Hong Kong. Specifically, the study sought to compare the effects of horticultural activities versus regular continuous monitoring on depressive symptoms, anxiety, self-esteem and quality of life among youth in Hong Kong. This study adheres to the CONSORT guidelines for transparent reporting [28].

### Trial design

This study employed an exploratory RCT design with an intervention group and a control group. A total of 108 participants were equally and randomly assigned to each group. Data about participants’ depressive symptoms, anxiety symptoms, self-esteem and quality of life were collected at three timepoints for both groups: baseline (T_0_), immediately after the intervention (T_1_) and 4 weeks post-intervention (T_2_), all through face-to-face assessments.

## Trial registration

This study is registered at the Chinese Clinical Trial Registry on 8 September 2023 with the trial registration number: ChiCTR2300075575.

## Participants

### Inclusion and exclusion criteria

A total of 108 Hong Kong youth aged 15 to 24 were recruited for this study. Eligibility was determined using the Patient Health Questionnaire-9 (PHQ-9), a validated tool for assessing the severity of depressive symptoms based on DSM-5 criteria [29–30]. Participants scoring between 5 and 19 on the PHQ-9—indicating mild to moderate depressive symptoms—were included. Individuals scoring 20 or above, which suggests severe depressive symptoms, were excluded and advised to seek professional medical support.

To ensure effective communication and smooth implementation of the intervention, only Cantonese-speaking individuals capable of following simple instructions were eligible. For safety reasons, individuals with known allergies to sunlight or vegetation were excluded. Additionally, owing to the physical nature of horticultural activities, those with cardiopulmonary conditions or other limitations preventing moderate physical exertion were not eligible.

To minimise confounding factors, individuals with diagnosed psychological disorders or those taking psychoactive medications were also excluded.

### Recruitment

Participants were recruited through social media platforms (Facebook and Instagram) and the distribution of flyers. The flyers were distributed at 10 locations near secondary schools and universities. Interested individuals completed a Google Form and provided their contact information for follow-up demographic screening by the research team.

### Sample size

Sample size estimation was informed by a previous RCT conducted in Hong Kong that examined the effects of horticultural therapy on depressive symptoms among elderly nursing home residents [19], which recruited 96 participants. As such, in this study, accounting for an anticipated 10% attrition rate, a minimum of 107 participants was deemed necessary. To ensure equal group allocation, 108 participants were ultimately recruited.

### Randomisation and allocation concealment

Simple randomisation was conducted using the online tool RESEARCH RANDOMIZER [31]. Allocation concealment was automatically managed by the platform, ensuring that group assignments remained unbiased. The nurse responsible for participant recruitment had no influence on the randomisation process.

### Blinding

Given the nature of the intervention, the blinding of participants and interventors was not feasible and therefore not applied in this study.

### Intervention group

#### Intervention content

The intervention was developed by the research team based on prior studies [13, 16, 32] and adapted to local conditions, including the availability of recreational farmland, manpower and climate. Key components identified as effective in previous research were selected and tailored to fit the venue and facility-related constraints.

The programme consisted of group-based horticultural activities involving 10–15 participants per group, delivered over four consecutive weekly sessions. Each participant was assigned a personal plot of farmland, which was labelled and maintained throughout the programme. Core activities included session orientation, horticultural education, hands-on farming, closing rituals, farmer-led storytelling, harvesting and food preparation. The selected crops were romaine lettuce, crown daisy (Glebionis coronaria) and water spinach (Ipomoea aquatica).

#### Settings

To align with the principles of Kaplan’s ART, the intervention site was selected to provide an immersive, nature-rich environment conducive to mental restoration. Tai Po Living Farm was chosen for its suitability for recreational horticulture and the availability of services to care for the plants being grown by the participants outside of programme hours. Located in a rural village, the farm offered a visually expansive and tranquil setting that encouraged mental engagement with nature. The main cultivated crops on the farmland focus on edible vegetables commonly found in Hong Kong, such as tomatoes, lettuces and okra. The subtropical climate in Hong Kong enables farming even during winter times.

The site was well equipped with essential facilities, including farming tools, lavatories, an indoor multipurpose room, an open kitchen and resting areas. Its accessibility via minibus and train added to its convenience for the participants. The infrastructure allowed a wide range of activities, including horticultural education, preparation of meals based on the crops grown by the participants and communal dining.

#### Session 1

The first session began with an introduction and activity briefing, followed by a PowerPoint presentation covering basic plant care techniques such as crop rotation, cover cropping and mechanical pest control. This indoor segment provided a comfortable learning environment and helped participants mentally prepare for the outdoor tasks.

Participants then transplanted mature seedlings into their assigned plots (approximately 1.5 × 1 m), guided by a farmer and a nurse. Lessons on sowing, fertilising and watering followed. Breaks were scheduled between activities. The session concluded with tool cleaning and debriefing by the nurse. During the debriefing period, the nurse gathered participants in a circle to provide psychological support, acknowledge their efforts and summarise the day’s activities.

#### Session 2

After orientation, participants observed the growth of their seedlings. The farmer discussed plant performance in relation to the weather, human activity and pests. This session emphasised themes such as accepting failure, managing perfectionism and challenging negative thoughts. Participants explored reasons for plant failure, categorised into controllable and uncontrollable factors, which were then metaphorically linked to life challenges [33].

Drawing on the work of Monroe [34], the nurse used horticultural metaphors to encourage self-reflection. For example, the resilience of plants in adverse conditions symbolised youths’ capacity to adapt to personal struggles. Routine farming tasks—watering, weeding and fertilising—were completed, followed by a break and the same session conclusion as described above.

#### Session 3

The third session began with crop maintenance, including weeding and irrigation. After a break, the farmer led a guided tour of the farm, showcasing areas such as the greenhouse, irrigation system and crop zones. In line with ART [23, 24], this exposure to natural scenery was intended to promote mental relaxation and recovery from attention fatigue. The session concluded with tool cleaning and debriefing.

#### Session 4

The final session began with orientation and crop harvesting [35]. Harvested crops were weighed to help the participants quantify their achievements and foster a sense of accomplishment. The participants then prepared pizzas using their produce, baked in a traditional wood- and charcoal-fired brick oven. They could enjoy the pizza on site or take it home. All participants confirmed that they had no known food allergies prior to this session.

As with previous sessions, the day ended with tool cleaning and a debriefing. Individual contributions were recognised to promote self-esteem and a sense of achievement. The nurse also facilitated self-reflection using open-ended questions to help the participants identify personal strengths demonstrated during the programme. The participants were encouraged to share their thoughts and emotions.

A detailed set of intervention protocol is provided in Table 1.

**Table 1:** Intervention protocol for horticultural activities programme

Activities	Session 1	Session 2	Session 3	Session 4
Briefing session (5–10 min)	• Attendance taking • Session activity orientation			
Activity 1	Education session (15 min)	Farming chores (30–35 min)	Farming chores (30–35 min)	Harvesting (20 min)
Content	1. Teaching organic farming ideas: - Crop rotation, cover cropping, organic pest control 2. Introduction of organic farming and conventional farming 3. Program content introduction	1. Weeds removal 2. Bug removal 3. Stabilising the crops’ positions if needed 4. Transplantation 2nd batch of more mature seedlings if the 1st batch failed	1. Weeds removal 2. Bug removal 3. Stabilising the crops’ positions if needed 4. Fertilisation	1. Weighting of crops 2. Cleaning harvested food
Rationale/ Theme	Rationale: An orientation is essential for psychologically preparing participants for the coming farm work	Rationale: Allow participants the opportunity to observe the transplanted seedlings and prepare for the following discussion session	Rationale: Plant care to facilitate harvest in the next session. Promote the expectancy of participants	Rationale: Quantifying harvest allows visualisation of participants’ contribution. Thus, to create a sense of achievement
Activity 2	Farming chores (30–35 min)	Horticultural metaphors and farmer experience sharing (20 min)	Sightseeing tour	Acknowledgement of participants’ effort (10 min)
Content	1. Each participant was assigned with 1.5 × 1 m growing plots 2. Weeds removal 3. Bug removal 4. Soil fertilisation	1. Discussion of the relationship between plant growth, weather, fertiliser usage and pests 2. Classification of the reasons for plant growth failure into controllable and uncontrollable 3. Relate plant growth conditions as obstacles that youth faced in life	1. A walking tour - watery system - Fruit planting zone - Vegetable zones 2. Education about crops was provided by the farmer during the walk	1. Promote self-esteem and a sense of achievement 2. Facilitated self-reflection using open-ended questions 3. Help participants identify personal strengths
Rationale/ Theme	Rationale: Growing plots assignment ensures each participant can perform hands-on practice and not be left out as an observer or bystander	Theme: accepting failure, managing perfectionism and challenging negative thoughts	Rationale: exposure to natural scenery was intended to promote mental relaxation and recovery from attention fatigue	Rationale: Psychologically provide positive feedback to participants, to boost self-esteem
Break (10 min)	Allow rest			
Activity 3	Farming chores (30–35 min)	Farming chores (30–35 min)	Farming chores (30–35 min)	Making pizzas from the harvest and lunch (40–50 min)
Content	1. Seedlings transplantation 2. Irrigation	Fertilisation to boost fruiting.	1. Weeds removal 2. Bug removal	1. Make pizza using a native stove built from bricks 2. Enjoy the pizza
Rationale/ Theme	Rationale: Continue unfinished horticultural tasks	Rationale: Continue unfinished horticultural tasks	Rationale: Continue unfinished horticultural tasks	Rationale: Sense of happiness created
Debriefing (10–15 min)	• Cleaning up of used utensils • Participants gathered in a circle to provide psychological support, acknowledge their efforts and summarise the day’s activities.			

#### Intervenors

Two intervenors facilitated the programme. A registered nurse was responsible for participant safety, intervention quality assurance and overall coordination. Given her prior experience in rooftop farming and indoor horticulture, the nurse also assisted with farming tasks and provided psychological support throughout the sessions.

A certified organic farmer who also owned the farm served as the second intervenor. Holding an advanced certificate in organic agriculture and a certificate in organic crop inspection, he was responsible for delivering farming instruction, sharing personal experiences and caring for the plants between the weekly sessions.

#### Intervention fidelity

Multiple strategies were employed to ensure the integrity and consistency of intervention implementation. First, a standardised program checklist detailing all individual activities and their designated durations was developed prior to the commencement of the program. All intervenors adhered strictly to the intervention protocol.

One month before the first intervention group commenced, the researcher visited the farm to brief the participating farmer and confirm his understanding of the procedures. A copy of the program checklist was provided. During all intervention sessions, the researcher monitored adherence by comparing activities against the checklist and reminding the farmer to avoid deviations.

Additionally, during the farming experience-sharing sessions, the farmer was instructed to present the same experience consistently across all four intervention batches to minimise variability in delivery.

### Control Group

The control group received regular continuous monitoring of depressive and anxiety symptoms, self-esteem and quality of life levels, throughout the programme. In addition, participants were provided with a Chinese version of a mental health information pamphlet developed by the Centre for Health Protection of the Department of Health, Government of the Hong Kong Special Administrative Region, which included details of professional counselling hotlines.

## Outcomes

### Feasibility and applicability of the study

To assess the feasibility and applicability of the study, several indicators were evaluated: eligibility rate, recruitment rate, retention rate, attendance rate and the appropriateness of both the assessment tools and the content of the intervention. At T_2_, participants in the intervention group were interviewed face-to-face to gather feedback on the comprehensiveness of the programme, their willingness to participate in future sessions and any additional comments. Any harm or adverse events were to be reported to the ethics committee in accordance with institutional guidelines.

### Depressive symptoms

Depressive symptoms were measured using the Chinese version of the Patient Health Questionnaire-9 [PHQ-9; 36–37], which assesses the severity and frequency of nine depressive symptoms based on DSM-5 criteria. Each item is rated in terms of frequency on a 4-point scale (0 to 3 scored on each item): “not at all,” “on some days,” “on more than half of days” and “nearly every day.” Total scores range from 0 to 27, with severity categorised as follows: minimal (0–4), mild (5–9), moderate (10–14), moderately severe (15–19) and severe (20+).

### Anxiety symptoms

Given the high comorbidity between depression and anxiety [38], anxiety symptoms were included as a secondary outcome. These were assessed using the Chinese version of the Generalized Anxiety Disorder-7 scale [GAD-7; 39–40]. GAD-7 evaluates seven anxiety symptoms on a scale of 0–3, with total scores ranging from 0 to 21. Severity is categorised as mild (0–4), moderate (5–9), moderately severe (10–14) and severe (15+).

### Self-esteem

Self-esteem was assessed using the Chinese version of the Rosenberg Self-Esteem Scale [RSE; 41–42], given its relevance to youth mental health and suicide risk [43]. RSE consists of 10 items rated on a 3-point Likert scale, with total scores ranging from 0 to 30. Scores are interpreted as representing low (0–14), moderate (15–25) and high self-esteem (26–30).

### Quality of life

Quality of life was measured using the Chinese version of the EuroQol 5-Dimension 5-Level instrument [(EQ-5D-5 L; 44–45]. Results are presented on a visual analogue scale ranging from 0 to 100, with higher scores indicating better perceived quality of life. No standardised cut-offs were applied.

### Data analysis

Feasibility indicators—including recruitment rate, eligibility rate, retention rate, attendance rate and appropriateness of assessment tools and intervention content—were evaluated using recruitment metrics and face-to-face interviews.

For psychological outcome measures, the data were analysed using an intention-to-treat approach. If missing data accounted for less than 10% of the dataset, repeated measures ANOVA was applied. If missing data exceeded this threshold, a GEE model was used to account for missingness and repeated measures. Additionally, demographic differences between participants who completed the study and those who withdrew were analysed to assess potential bias.

## Results

### Participant flow

**Fig. 1:**
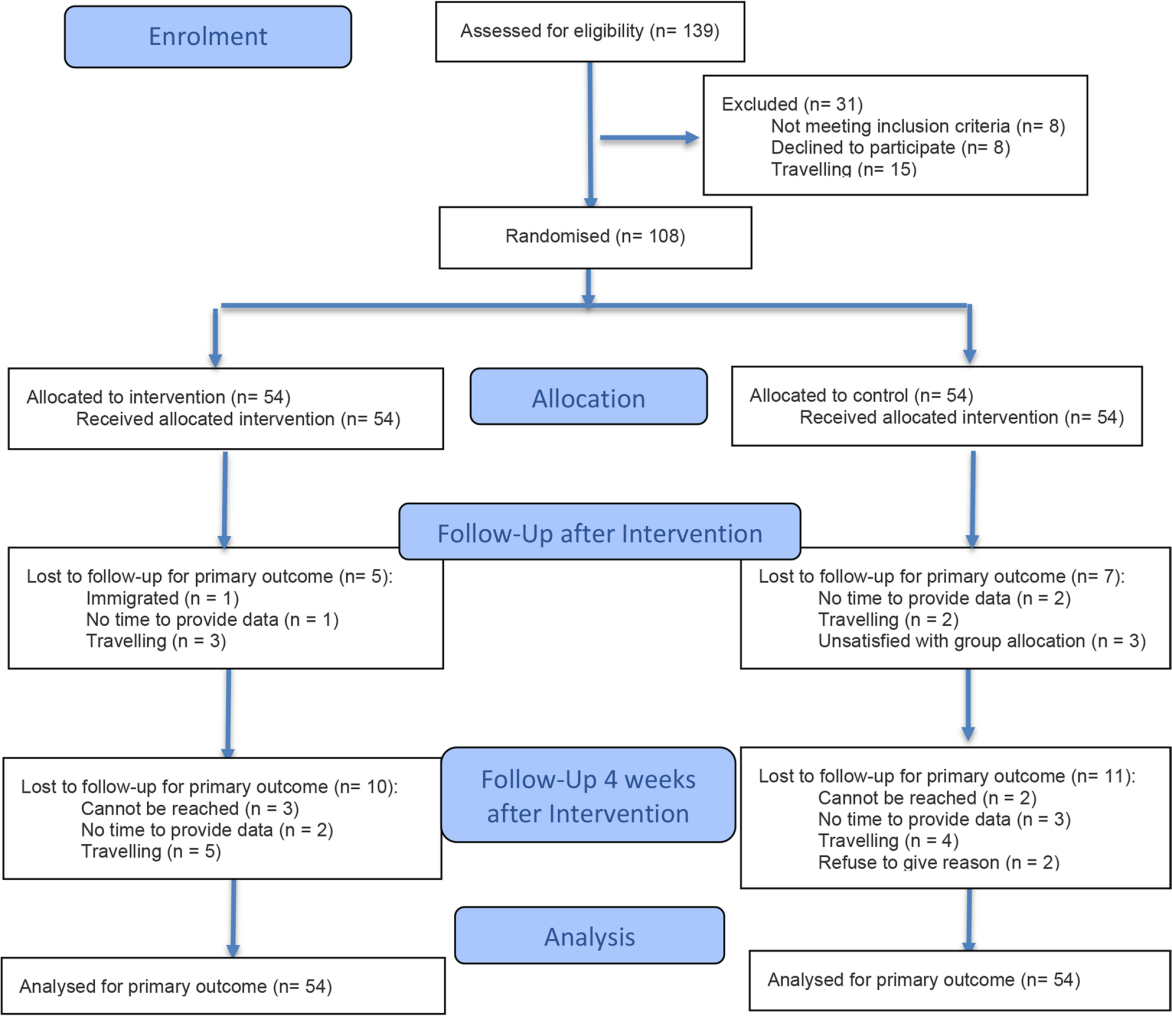
Participant flow chart

Figure 1 illustrates the participant flow throughout the study. Between October and December 2023, 139 individuals completed the online self-recruitment form. Of these, 131 met the inclusion criteria. Among the eligible participants, 8 declined to sign the consent form and 15 were unable to enrol as a result of scheduling conflicts due to a trip. The remaining 108 participants were enrolled and randomly assigned to either the intervention group (*n* = 54) or the control group (*n* = 54).

Four batches of the intervention were conducted between October 2023 and January 2024, with final data collection completed in March 2024. No deviations from the protocol or adverse events were reported. Overall, 96 participants remained at the post-intervention stage, while 75 completed the 4-week follow-up. The control group experienced a slightly higher attrition rate (27.8% dropout in the intervention group and 33.3% dropout in the control group, *P* = .53).

### Baseline characteristics

Table 2 presents the baseline demographic and psychological characteristics of the participants, including age, sex, education level, and initial scores on depressive symptoms and other psychological measures. No statistically significant differences were observed between the intervention and control groups at baseline

**Table 2:** Demographic data of participants

	Total	Intervention Group (*n* = 54)	Control Group (*n* = 54)	X^2^ (df) or t (df)	*p*-value
Gender (total)	108 (100%)				
Male	38 (35.2%)	20 (37.0%)	18 (33.3%)		
Female	70 (64.8%)	34 (63.0%)	36 (66.7%)	0.162 (1)	0.840
Age: Mean (SD)	21.43 (2.669)	21.07(2.847)	21.78 (2.455)	1.376 (106) **	0.172
Educational Level					
Middle School	2 (1.9%)	2 (3.7%)	0 (0%)		
High School	26 (24.1%)	14 (25.9%)	12 (22.2%)		
University	80 (74.1%)	38 (70.4%)	42 (77.8%)	1.995 *	0.455
Main Language					
Cantonese	108 (100%)	54 (100%)	54 (100%)	1.00	1.000
English	3 (2.8%)	3 (5.5%)	0 (0%)	N/A *	0.243
Mandarin	2 (1.9%)	2 (3.7%)	0 (0%)	N/A *	0.495
Occupation					
Student	65 (60.2%)	34 (63.0%)	31 (57.4%)		
Unemployment	4 (3.7%)	2 (3.7%)	2 (3.7%)		
Part-time	15 (13.9%)	7 (13.0%)	8 (14.8%)		
Full-time	24 (22.2%)	11 (20.4%)	13 (24.1%)	0.540 *	0.942
Religion					
None	96 (88.9%)	47 (87.0%)	49 (90.7%)		
Christianity	10 (9.3%)	6 (11.1%)	4 (7.4%)		
Others	2 (1.9%)	1 (0.9%)	1 (0.9%)	0.698 *	0.870
Psychological Parameters					
Depressive symptoms	10.11 (3.556)	10.24 (3.706)	9.98 (3.428)	-0.377 (106) **	0.707
Anxiety symptoms	7.94 (3.591)	7.81 (3.234)	8.07 (3.942)	0.374 (106) **	0.709
Self-esteem	26.24 (4.053)	26.13 (3.497)	26.35 (4.573)	0.284 (106) **	0.777
Quality-of-life	72.41 (19.294)	73.66 (17.282)	71.17 (21.207)	-0.669 (106) **	0.503

*Fisher-Freeman-Halton Exact test performed due to > 20% cells having an expected cell count less than 5

**A 2-sample t-test was performed

### Feasibility and applicability

The study demonstrated strong feasibility. The eligibility rate was 94.2% (131/139), the recruitment rate was 77.7% (108/131), and the retention rates were 88.9% (96/108) at post-intervention (T_1_) and 69.44% (75/108) at 4-week follow-up (T_2_). The most common reason for dropping out of the study was travel, particularly during public holidays. At T_2_, retention rates were 72.2% (39/54) in the intervention group and 66.7% (36/54) in the control group.

The highest absence rate occurred during Session 3, accounting for 25.9% of all absences. However, over 90% of participants attended at least three sessions, which covered the core components of the intervention, indicating a high completion rate. Notably, only one participant was recruited via flyer distribution; the rest were recruited online.

All participants reported that the questionnaires were easy to understand and could be completed within 5–10 min. During the post-intervention interviews, the participants in the intervention group provided positive feedback. Many appreciated the simplicity and accessibility of the activities, with comments such as, “I originally thought we would have to work hard like farmers on TV” and “The activities are okay for beginners. Girls like me can easily perform them.” The low physical and technical demands of the programme were seen as facilitators, making it accessible even to those without previous experience of horticulture. The participants enjoyed tasks such as weeding and transplanting seedlings. The participants also expressed an interest in future participation in similar horticultural projects. One participant reported starting a gardening hobby with four others after the programme. All interviewees indicated a willingness to receive updates about similar activities via mobile apps. However, 14 of the 33 dropouts were due to travel during public holidays, suggesting that scheduling around holidays may have been a barrier to participation.

### Psychological parameters

Given that the attrition rate exceeded 10%, GEE analysis was used to evaluate the intervention’s effects on psychological outcomes (Table 3). The intervention group showed a statistically significant reduction in depressive symptoms at T_1_ (β = −1.207, 95% CI: −1.820 to − 0.043, *P* = .028). However, this effect showed only a tendency for significance at T_2_ (*β* = −0.932, 95% CI: −2.233 to − 0.180, *P* = .061).

**Table 3:** Generalised estimation equation analysis of dependent outcomes

Outcome	Estimated Marginal Mean (SE)		Group X Time Beta (SE)	Unstandardized Mean Difference 95% Confidence Interval	Test of Model Effect			Interaction Across Timepoints	95% WALD Confidence Interval
Depressive symptom	IG	CG			Group effect	Time effect	Group X time effect		
T0	10.27 (0.492)	10.23 (0.483)							
T1	8.58 (0.435)	9.75 (0.434)	−1.207 (0.5236)	−1.820 – −0.043				T1: *p* = .050	−0.33–0.00
T2	8.78 (0.409)	9.67 (0.502)	−0.932 (0.4532)	−2.233 – −0.180				T2: *p* = .610	−2.13–0.35
					*p* = .231	*p* < .001	*p* = .028		
Anxiety symptoms	IG	CG							
T0	7.81 (0.425)	8.20 (0.528)							
T1	7.03 (0.380)	8.04 (0.495)	−0.623 (0.7019)	−1.999–0.753				T1: *p* = .109	−2.24–0.23
T2	7.52 (0.333)	8.24 (0.492)	−0.335 (0.7245)	−1.755–1.086				T2: *p* = .231	−1.89–0.46
					*p* = .173	*p* = .256	*p* = .646		
Self-esteem	IG	CG							
T0	26.09 (0.438)	26.14 (0.661)							
T1	28.23 (0.513)	27.06 (0.595)	1.228 (0.6942)	−0.343 — 2.793				T1: *p* = .134	−0.36–2.71
T2	28.62 (0.450)	27.45 (0.746)	1.225 (0.8001)	−1.33 — 2.589				T2: *p* = .176	−0.53–2.88
					*p* = .260	*p* < .001	*p* = .1441		
Quality of life	IG	CG							
T0	73.70 (2.326)	69.97 (2.862)							
T1	79.04 (1.663)	70.72 (2.532)	4.592 (2.3050)	0.0253–9.110				T1: *p* = .005	−2.45–14.20
T2	80.05 (1.740)	71.08 (2.673)	5.238 (2.8141)	−0.277–10.754	*p* = .016	*p* = .010	*p* = .084	T2: *p* = .004	2.82–15.13

No statistically significant changes were observed in anxiety symptoms, self-esteem or quality of life at any timepoint (all *P* > .05). The group × time interaction effects were also non-significant for anxiety (*P* = .65), self-esteem (*P* = .14) and quality of life (*P* = .08). Nonetheless, all psychological parameters showed greater numerical improvements in the intervention group than in the control group, despite the lack of statistical significance. Based on Cohen’s guidelines [46], the effect size for depressive symptom reduction at T_1_ was small to moderate (Cohen’s *d* = − 0.34).

### Missing data analysis

As shown in Table 4, participants with lower baseline depressive symptoms were more likely to drop out of the study (*P* = .047), suggesting a potential bias in attrition.

**Table 4:** Missing data analysis

	Total	Retained Participants (*n* = 75)	Dropout (*n* = 33)	X^2^ (df) or t (df)	*p* value
Gender (total)	108 (100%)				
Male	38 (35.2%)	27 (36.0%)	11 (33.3%)		
Female	70 (64.8%)	48 (64.0%)	22 (66.7%)	0.071 (1)	0.830
Age: mean (SD)	21.43 (2.669)	21.36 (2.700)	21.58 (2.634)	0.385 (106) **	0.699
Educational level					
Middle school	2 (1.9%)	1 (1.3%)	1 (3.0%)		
High school	26 (24.1%)	18 (24.0%)	8 (24.2%)		
University	80 (74.1%)	56 (74.7%)	24 (72.7%)	0.765 *	0.908
Main language					
Cantonese	108 (100%)	75 (100%)	33 (100%)	1.00	1.000
English	3 (2.8%)	2 (2.7%)	1 (3.0%)	NA*	1.000
Mandarin	2 (1.9%)	2 (2.7%)	0 (0.0%)	NA*	1.000
Occupation					
Student	65 (60.2%)	45 (60.0%)	20 (60.6%)		
Unemployment	4 (3.7%)	2 (2.7%)	2 (6.1%)		
Part-time	15 (13.9%)	11 (14.7%)	4 (12.1%)		
Full-time	24 (22.2%)	17 (22.7%)	7 (21.2%)	1.048*	0.854
Religion					
None	96 (88.9%)	68 (90.7%)	28 (84.8%)		
Christianity	10 (9.3%)	7 (9.3%)	3 (9.1%)		
Others	2 (1.9%)	0 (0.0%)	2 (6.1%)	3.878*	0.151
Psychological Parameters					
Depressive symptoms	10.11 (3.556)	10.56 (3.538)	9.09 (3.431)	−2.006 (106) **	0.047
Anxiety symptoms	7.94 (3.591)	8.39 (3.353)	6.94 (3.952)	−1.955 (106) **	0.053
Self-esteem	26.24 (4.053)	25.83 (3.825)	27.18 (4.447)	1.613 (106) **	0.110
Quality of life	72.41 (19.294)	70.17 (18.765)	77.52 (19.794)	1.844 (106) **	0.068

*Fisher-Freeman-Halton Exact test performed due to > 20% cells having expected cell count less than five

**A two-sample t-test was performed

## Discussion and interpretation

This RCT addresses a significant gap in the literature by exploring the impact of horticultural activities on depressive symptoms among Chinese youth—an area that remains under-researched. As one of the first studies of its kind in Hong Kong, this work offers original insights into the feasibility, acceptability and preliminary effectiveness of horticultural interventions for promoting mental well-being in this population.

### Effect size

This study found that horticultural activities significantly reduced depressive symptoms among Hong Kong youth, with a small effect size (Cohen’s d = − 0.34). While this is a promising result, it is smaller than the effect size reported in a recent meta-analysis of horticultural interventions for community-dwelling adults [14; SMD = − 0.67]. For secondary outcomes—anxiety symptoms, self-esteem and quality of life—the estimated effect sizes were also small (Cohen’s d = − 0.17, − 0.30 and − 0.24, respectively) and did not reach statistical significance.

These findings are broadly consistent with previous studies exploring horticultural interventions for youth, including the previously mentioned research [15–17], which reported varying effect sizes (SMDs ranging from − 1.03 to 0.56). However, differences in study design, sample characteristics and intervention content may explain the variation in outcomes. For example, Ghanbari et al. [16] focused on 50 Iranian female students with clinically diagnosed depression, while Curzio et al. [17] conducted a pilot study with 12 Italian female youths with anorexia nervosa. Meanwhile, Buru et al. [15] included only 16 university students from South Korea, and limited details were provided about the content of the intervention. In contrast, the present study targeted a broader population of Hong Kong youth with mild to moderate depressive symptoms using a standardised, community-based horticultural programme.

The smaller-than-expected effect size may also reflect cultural and contextual differences. As this was one of the first studies of its kind in Hong Kong, there were limited local precedents to guide the design of the intervention. The programme was developed based on Kaplan’s ART and informed by the general principles outlined in a meta-analysis of 30 studies on the effects of horticultural activities for reducing depressive symptoms in adults by Sin et al. [14], which may not fully align with the preferences or needs of youth in Hong Kong. Cultural attitudes towards nature-based interventions and mental health may also influence engagement and outcomes.

Additionally, the relatively small sample size (*n* = 108) and high attrition rate at T_2_ (30.56%) may have reduced the statistical power needed to detect significant effects, particularly for secondary outcomes. The mean baseline PHQ-9 score in the intervention group was 10.24, indicating that most participants had only mild depressive symptoms. This may have introduced a ceiling effect, limiting the potential for measurable improvement.

### Feasibility and applicability

The study demonstrated strong feasibility. The high eligibility (94.2%) and recruitment (77.7%) rates suggest that the intervention was accessible and appealing to the target population. Reaching the recruitment target within 3 months indicates that the programme resonated with local youth. The inclusion criteria were also found to be appropriate and the intervention was well received.

The use of brief, self-administered questionnaires also proved effective, with all participants able to complete them within 5–10 min. The Likert scale and numeric formats facilitated straightforward data collection and analysis. However, relying solely on in-person data collection posed logistical challenges, particularly at T_2_, when many participants were unavailable due to travel. This contributed to the higher attrition rate and suggests that incorporating online or hybrid follow-up methods may improve retention in future studies.

The participants responded positively to the content of the intervention. Many appreciated the manageable difficulty level of the farming tasks and expressed interest in participating in similar projects in future. The fact that several participants started a gardening hobby after the programme suggests that the intervention was not only feasible but also engaging and impactful. However, attendance was notably lower during Session 3, which featured a guided farm walk and fewer hands-on activities. This suggests that more interactive components may be necessary to maintain engagement throughout the programme.

The implementation of the intervention by a nurse and a certified farmer on a recreational farm was both practical and well received. The participants were willing to travel to rural areas for weekend sessions, and the physical demands of the activities were appropriate for youth. The timing of the intervention—from October to January—was also suitable given Hong Kong’s subtropical climate, with cold-resistant crops being used.

### Generalisability

The sample included a higher proportion of female participants (64.8%), which aligns with global trends showing that females are approximately twice as likely to experience depressive disorders as males [47]. This enhances the generalisability of the findings to the broader population of youth affected by depression. Additionally, the use of social media for recruitment helped minimise geographical bias, resulting in a relatively diverse sample across Hong Kong.

However, the ratio of school-age to university-age participants was not balanced, which may limit the applicability of the findings to younger teenagers below 18 years old. Future studies should consider stratified recruitment strategies to ensure the representation of a broader range of age groups.

### Strengths

This study employed a rigorous RCT design to evaluate the effects of horticultural activities on depressive symptoms among Hong Kong youth. As one of the first local studies in this area, it provides valuable evidence on the feasibility and potential of nature-based interventions for this population.

Intervention fidelity was ensured through a detailed programme manual, pre-intervention training for the farmer and ongoing quality monitoring by the researcher using a structured checklist. These measures promoted consistency across sessions and enhanced the reliability and replicability of the findings.

The intervention was also resource-efficient. Delivered by a nurse and a farmer—without the involvement of psychiatrists or clinical psychologists—it minimised costs and avoided burdening the public healthcare system. In addition, its group-based format allowed a high participant-to-intervenor ratio, making it both time- and manpower-efficient.

### Limitations and possible improvements

Several limitations of this study should be acknowledged. First, as with many behavioural interventions, the findings may have been influenced by methodological biases, including the Hawthorne effect, self-selection bias, and social desirability bias. In addition, the absence of an independent, blinded outcome assessor may have introduced observer-expectancy effects. Psychological outcomes were measured using self-reported questionnaires, such as the PHQ‑9, which rely on participants’ recall of symptoms over the preceding two weeks. This approach may be subject to reporting bias, including symptom overestimation or underestimation. Future studies could strengthen methodological rigor by incorporating blinded assessors, multi-method outcome measurement (e.g. clinician-rated scales or ecological momentary assessment), and objective indicators where feasible.

Second, the intervention was delivered exclusively in an outdoor setting, which may have limited participation among youth with physical disabilities, dermatological conditions, allergies, or environmental sensitivities. Moreover, the outdoor, group-based nature of the programme may have preferentially attracted individuals who were already more receptive to outdoor activities or who possessed a more outgoing or socially engaged disposition. As a result, the intervention may have been less appealing to youth with introverted traits or a preference for indoor environments, potentially limiting the representativeness of the sample. To address this concern, future trials should consider indoor, hybrid, or home-based horticultural formats (e.g. indoor pot gardening, greenhouse activities, or digital-supported gardening programmes) to accommodate diverse preferences and functional abilities.

Third, the relatively modest sample size constrained statistical power and may have limited the detection of significant effects, particularly for secondary psychological outcomes. In addition, participants with lower baseline depressive symptom severity were more likely to withdraw during follow-up, suggesting a potential floor effect. Future studies may benefit from applying a slightly higher inclusion threshold (e.g. PHQ‑9 ≥ 8) to better target youth at risk while reducing attrition.

From a generalisability perspective, several limitations warrant consideration. The study sample comprised youth aged 15–24 years and excluded individuals with severe depressive symptoms (PHQ‑9 ≥ 20) for ethical and safety reasons. Consequently, the findings cannot be generalised to younger children, older adults, or youth with severe or clinically diagnosed depression. Moreover, participants were recruited on a self-selected basis, which may have resulted in a sample that was more motivated, health-conscious, or interested in horticultural activities at baseline, potentially inflating observed intervention effects.

Geographically, the sample was drawn exclusively from Hong Kong, a densely populated, high-income, and culturally specific urban setting. Cultural norms related to mental health stigma, family structure, and attitudes toward nature-based activities may differ substantially across regions. As such, caution is required when extrapolating the findings to other cultural or geographical contexts. Replication studies in other Asian regions and Western countries are needed to assess cross-cultural applicability.

Finally, attrition at the 4‑week follow-up (T2), primarily due to travel during public holidays, highlights the logistical challenges associated with in-person data collection. Future studies could improve retention by incorporating online or hybrid follow-up assessments, increasing scheduling flexibility, and reducing participant burden.

### Implications and suggestions for future research

To build on the findings of this exploratory study, a full-scale RCT with a larger sample should be conducted to enhance statistical power and generalisability. Extending the duration of the intervention is also recommended, as the short-term nature of the current programme may have limited its sustained impact. An 8-week programme with interim assessments could better capture therapeutic effects and explore dose–response relationships.

Implementing horticultural activities within school or university settings may improve accessibility and reduce logistical barriers. Additionally, exploring indoor or virtual formats—such as greenhouse-based sessions or online pot gardening—could increase inclusivity for individuals with physical limitations or environmental sensitivities.

The findings of this study provide preliminary evidence that ART is both applicable and acceptable among Chinese youth, with the horticultural intervention demonstrating satisfactory effectiveness and feasibility. These results suggest that combining horticultural activities with an ART-based framework may represent a promising pathway for the early control and prevention of depressive symptoms in youth, particularly within non-clinical, community-based settings.

From a developmental perspective, ART-based horticultural interventions may help youth restore attentional capacity, reduce cognitive fatigue, and improve emotional regulation during a life stage characterised by intense academic, social, and identity-related demands. Importantly, such interventions can be delivered outside formal healthcare systems, offering a scalable and low-cost complement to existing mental health services.

At the family level, future research should explore family-centred applications of ART-informed horticultural activities. For example, home gardening, rooftop gardening, or community allotment gardening may facilitate shared restorative experiences among family members, enhance parent–child interaction, and reduce intergenerational stress. These family-based approaches may be particularly valuable in collectivistic cultures, where family involvement plays a central role in youth development and well-being. Investigating the psychological benefits of intergenerational horticultural engagement may further extend the preventive potential of ART beyond the individual to the family system.

Future studies are encouraged to examine longer intervention durations, family-based or dyadic designs, and the integration of school-, community-, and home-based horticultural programmes. Such directions would help to clarify the role of ART-informed horticultural activities as a sustainable, culturally sensitive, and developmentally appropriate mental health strategy for youth and their families.

## Conclusion

This exploratory RCT demonstrated that horticultural activities can effectively reduce depressive symptoms among Hong Kong youth, with a small but meaningful effect size. The intervention was well received, low-cost and resource-efficient, with high feasibility and acceptability confirmed through participant feedback and implementation outcomes.

Although our findings are promising, further research is needed to refine the intervention, increase sample size, extend programme duration, and improve retention. With continued development, horticultural activities hold strong potential as a scalable, community-based mental health strategy for youth.

## Supplementary Information

Supplementary Material 1

Supplementary Material 2

## Data Availability

The datasets used and/or analysed during the current study are available from the corresponding author on reasonable request.
