# Supplementary material for: Nurse‑led horticultural activities as an early intervention for mild‑to‑moderate depressive symptoms among youth in Hong Kong: an exploratory randomised controlled trial
Source: BMC Public Health. 2026 Apr 14;26:1676. doi: 10.1186/s12889-026-27376-3 (PMC13195863; doi:10.1186/s12889-026-27376-3)
Supplement: Supplementary file 1 — Supplementary Material 1 [file 12889_2026_27376_MOESM1_ESM.pdf]

Subject administered Questionnaire: Patient Health Questionnaire-9 (Version 1) Date: 07/06/2023

病人健康狀況問卷-9

在過去兩個星期，你有多經常受以下問題困擾？

1. 做任何事都覺得沉悶或者根本不想做任何事

☐ 完全不會 ☐ 幾天 ☐ 一半以上的天數 ☐ 近乎每天

2. 情緒低落、抑鬱或絕望

☐完全不會 ☐ 幾天 ☐ 一半以上的天數 ☐ 近乎每天

3. 難於入睡；半夜會醒或相反地睡覺時間過多

☐完全不會 ☐ 幾天 ☐ 一半以上的天數 ☐ 近乎每天

4. 覺得疲倦或活力不足

☐完全不會 ☐ 幾天 ☐ 一半以上的天數 ☐ 近乎每天

5. 胃口極差或進食過量

☐完全不會 ☐ 幾天 ☐ 一半以上的天數 ☐ 近乎每天

6. 不喜歡自己----覺得自己做得不好、對自己失望或有負家人期望

☐完全不會 ☐ 幾天 ☐ 一半以上的天數 ☐ 近乎每天

7. 難於集中精神做事，例如看報紙或看電視

☐完全不會 ☐ 幾天 ☐ 一半以上的天數 ☐ 近乎每天

8. 其他人反映你行動或說話遲緩；或者相反地，你比平常活動更多 ---- 坐立不安、停不下來

☐完全不會 ☐ 幾天 ☐ 一半以上的天數 ☐ 近乎每天

9. 想到自己最好去死或者自殘

☐完全不會 ☐ 幾天 ☐ 一半以上的天數 ☐ 近乎每天

本問卷由 Robert L. Spitzer 博士、Janet B.W. Williams 博士、Kurt Kroenke 博士和同事用 Pfizer Inc.提供的教育基金設計。無需准許即可複製、翻譯、展示或分發。

焦慮程度評估問卷-7

在過去兩個星期，你有多經常受以下問題困擾？

1. 感到緊張、不安或煩躁

☐ 完全不會 ☐ 幾天 ☐ 一半以上的天數 ☐ 近乎每天

2. 無法停止或控制憂慮

☐完全不會 ☐ 幾天 ☐ 一半以上的天數 ☐ 近乎每天

3. 過份憂慮不同的事情

☐完全不會 ☐ 幾天 ☐ 一半以上的天數 ☐ 近乎每天

4. 難以放鬆

☐完全不會 ☐ 幾天 ☐ 一半以上的天數 ☐ 近乎每天

5. 心情不寧以至坐立不安

☐完全不會 ☐ 幾天 ☐ 一半以上的天數 ☐ 近乎每天

6. 容易心煩或易怒

☐完全不會 ☐ 幾天 ☐ 一半以上的天數 ☐ 近乎每天

7. 感到害怕、就像要發生可怕的事情

☐完全不會 ☐ 幾天 ☐ 一半以上的天數 ☐ 近乎每天

本問卷由 Robert L. Spitzer 博士、Janet B.W. Williams 博士、Kurt Kroenke 博士和同事用 Pfizer Inc.提供的教育基金設計。
無需准許即可複製、翻譯、展示或分發。

羅森伯格自尊量表

請回想最近一個月來，發生下列各狀況的頻率。

1. 總括來說，我對自己感到滿意。

☐ 很同意 ☐同意 ☐ 不同意 ☐ 很不同意

2. 有些時候，我會覺得自己完全沒有用。

☐很同意 ☐同意 ☐ 不同意 ☐ 很不同意

3. 我感到自己是有一些優點。

☐很同意 ☐同意 ☐ 不同意 ☐ 很不同意

4. 我能夠把事情做得和大多數人一樣好。

☐很同意 ☐同意 ☐ 不同意 ☐ 很不同意

5. 我覺得自己沒有什麼值得自豪的地方。

☐很同意 ☐同意 ☐ 不同意 ☐ 很不同意

6. 有時我真的感到自己沒有用。

☐很同意 ☐同意 ☐ 不同意 ☐ 很不同意

7. 我感到自己是一個有價值的人，而我的價值起碼並不比別人低。

☐很同意 ☐同意 ☐ 不同意 ☐ 很不同意

8. 我希望我能夠對自己有更多的尊重。

☐很同意 ☐同意 ☐ 不同意 ☐ 很不同意

9. 總括來說，我傾向於感到自己像一個失敗者。

☐很同意 ☐同意 ☐ 不同意 ☐ 很不同意

10. 我抱著積極的態度面對自己。

☐很同意 ☐同意 ☐ 不同意 ☐ 很不同意

健康問卷

供香港地區使用之版本

(Traditional Chinese version for Hong Kong)

Hong Kong (Traditional Chinese) © 2012 EuroQol Group EQ-5D™ is a trade mark of the EuroQol Group

Subject administered Questionnaire: EQ-5D-5L (Version 1) Date: 07/06/2023

請在每個標題下剔選最能形容您<u>今天</u>的健康狀況的<u>一個</u>方格。

行動能力

我可以四處走動，沒有任何問題 ❑

我的行動有輕微問題 ❑

我的行動有中度問題 ❑

我的行動有嚴重問題 ❑

我無法行動 ❑

自我照顧

我在洗澡或穿衣方面沒有任何問題 ❑

我在洗澡或穿衣方面有輕微問題 ❑

我在洗澡或穿衣方面有中度問題 ❑

我在洗澡或穿衣方面有嚴重問題 ❑

我無法自己洗澡或穿衣 ❑

平常活動 (*如工作*、*讀書*、*家務*、*家庭或休閒活動*)

我能進行平常活動，沒有任何問題 ❑

我在進行平常活動方面有輕微問題 ❑

我在進行平常活動方面有中度問題 ❑

我在進行平常活動方面有嚴重問題 ❑

我無法進行平常活動 ❑

疼痛 / 不舒服

我沒有任何疼痛或不舒服 ❑

我覺得輕微疼痛或不舒服 ❑

我覺得中度疼痛或不舒服 ❑

我覺得嚴重疼痛或不舒服 ❑

我覺得極度疼痛或不舒服 ❑

焦慮 / 沮喪

我不覺得焦慮或沮喪 ❑

我覺得輕微焦慮或沮喪 ❑

我覺得中度焦慮或沮喪 ❑

我覺得嚴重焦慮或沮喪 ❑

我覺得極度焦慮或沮喪 ❑

Hong Kong (Traditional Chinese) © 2012 EuroQol Group EQ-5D™ is a trade mark of the EuroQol Group

- 我們想知道您今天的健康狀況有多好或多壞。
- 這個量度尺的**刻度**由 0 數到 100。
- 100 表示您能想像到的最好的健康狀況。

  0 表示您能想像到的最壞的健康狀況。
- 請在量度尺中打交叉，以顯示您今天的健康狀況如何。
- 現在，請在下面的方**格**中填寫您在**量度**尺上打叉的**刻度**。

您今天的健康狀況=

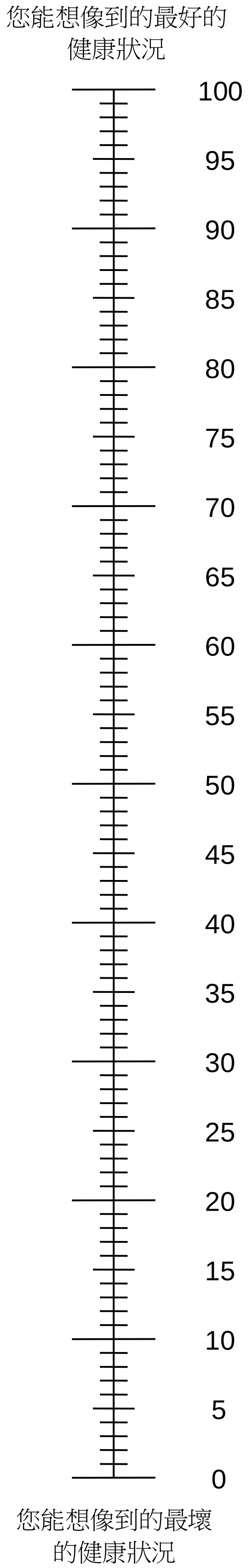

Hong Kong (Traditional Chinese) © 2012 EuroQol Group EQ-5D™ is a trade mark of the EuroQol Group
