# Supplementary material for: Nurse‑led horticultural activities as an early intervention for mild‑to‑moderate depressive symptoms among youth in Hong Kong: an exploratory randomised controlled trial
Source: BMC Public Health. 2026 Apr 14;26:1676. doi: 10.1186/s12889-026-27376-3 (PMC13195863; doi:10.1186/s12889-026-27376-3)
Supplement: Supplementary file 2 — Supplementary Material 2 [file 12889_2026_27376_MOESM2_ESM.pdf]

Patient Health Questionnaire-9

Over the last 2 weeks, how often have you been bothered by any of the following problems?

1. Little interest or pleasure in doing things

☐ Not at all ☐ Several days ☐ More than half the days ☐ Nearly every day

2. Feeling down, depressed, or hopeless

☐Not at all ☐ Several days ☐ More than half the days ☐ Nearly every day

3. Trouble falling or staying asleep, or sleeping too much

☐Not at all ☐ Several days ☐ More than half the days ☐ Nearly every day

4. Feeling tired or having little energy

☐Not at all ☐ Several days ☐ More than half the days ☐ Nearly every day

5. Poor appetite or overeating

☐Not at all ☐ Several days ☐ More than half the days ☐ Nearly every day

6. Feeling bad about yourself — or that you are a failure or have let yourself or your family down

☐Not at all ☐ Several days ☐ More than half the days ☐ Nearly every day

7. Trouble concentrating on things, such as reading the newspaper or watching television

☐Not at all ☐ Several days ☐ More than half the days ☐ Nearly every day

8. Moving or speaking so slowly that other people could have noticed? Or the opposite — being so fidgety or restless that you have been moving around a lot more than usual

☐Not at all ☐ Several days ☐ More than half the days ☐ Nearly every day

9. Thoughts that you would be better off dead or of hurting yourself in some way

☐Not at all ☐ Several days ☐ More than half the days ☐ Nearly every day

Generalized Anxiety Disorder 7-item

Over the last 2 weeks, how often have you been bothered by the following problems?

1. Feeling nervous, anxious or on edge

☐ Not at all ☐ Several days ☐ More than half the days ☐ Nearly every day

2. Not being able to stop or control worrying

☐Not at all ☐ Several days ☐ More than half the days ☐ Nearly every day

3. Worrying too much about different things

☐Not at all ☐ Several days ☐ More than half the days ☐ Nearly every day

4. Trouble relaxing

☐Not at all ☐ Several days ☐ More than half the days ☐ Nearly every day

5. Being so restless that it is hard to sit still

☐Not at all ☐ Several days ☐ More than half the days ☐ Nearly every day

6. Becoming easily annoyed or irritable

☐Not at all ☐ Several days ☐ More than half the days ☐ Nearly every day

7. Feeling afraid as if something awful might happen

☐Not at all ☐ Several days ☐ More than half the days ☐ Nearly every day

Rosenberg Self-Esteem Scale

Over the past month, how often have you experienced any of the following?

1. On the whole, I am satisfied with myself.
☐ Strongly agree ☐Agree ☐ Disagree ☐ Strongly disagree

2. At times I think I am no good at all
☐Strongly agree ☐Agree ☐ Disagree ☐ Strongly disagree

3. I feel that I have a number of good qualities.
☐Strongly agree ☐Agree ☐ Disagree ☐ Strongly disagree

4. I am able to do things as well as most other people.
☐Strongly agree ☐Agree ☐ Disagree ☐ Strongly disagree

5. I feel 1do not have much to be proud of.
☐Strongly agree ☐Agree ☐ Disagree ☐ Strongly disagree

6. I certainly feel useless at times.
☐Strongly agree ☐Agree ☐ Disagree ☐ Strongly disagree

7. I feel that I'm a person of worth.
☐Strongly agree ☐Agree ☐ Disagree ☐ Strongly disagree

8. I wish I could have more respect for myself.
☐Strongly agree ☐Agree ☐ Disagree ☐ Strongly disagree

9. All in all, I am inclined to think that I am a failure.
☐Strongly agree ☐Agree ☐ Disagree ☐ Strongly disagree

10. I take a positive attitude toward myself.
☐Strongly agree ☐Agree ☐ Disagree ☐ Strongly disagree

Health Questionnaire

English version for the UK

Sample

UK (English) © 2009 EuroQol Group EQ-5D™ is a trade mark of the EuroQol Group

Under each heading, please tick the ONE box that best describes your health TODAY.

MOBILITY

I have no problems in walking about ❑

I have slight problems in walking about ❑

I have moderate problems in walking about ❑

I have severe problems in walking about ❑

I am unable to walk about ❑

SELF-CARE

I have no problems washing or dressing myself ❑

I have slight problems washing or dressing myself ❑

I have moderate problems washing or dressing myself ❑

I have severe problems washing or dressing myself ❑

I am unable to wash or dress myself ❑

USUAL ACTIVITIES *(e.g. work, study, housework, family or leisure activities)*

I have no problems doing my usual activities ❑

I have slight problems doing my usual activities ❑

I have moderate problems doing my usual activities ❑

I have severe problems doing my usual activities ❑

I am unable to do my usual activities ❑

PAIN / DISCOMFORT

I have no pain or discomfort ❑

I have slight pain or discomfort ❑

I have moderate pain or discomfort ❑

I have severe pain or discomfort ❑

I have extreme pain or discomfort ❑

ANXIETY / DEPRESSION

I am not anxious or depressed ❑

I am slightly anxious or depressed ❑

I am moderately anxious or depressed ❑

I am severely anxious or depressed ❑

I am extremely anxious or depressed ❑

Sample

UK (English) © 2009 EuroQol Group EQ-5D™ is a trade mark of the EuroQol Group

- We would like to know how good or bad your health is TODAY.
- This scale is numbered from 0 to 100.
- 100 means the best health you can imagine.
  0 means the worst health you can imagine.
- Mark an X on the scale to indicate how your health is TODAY.
- Now, please write the number you marked on the scale in the box below.

YOUR HEALTH TODAY = ☐

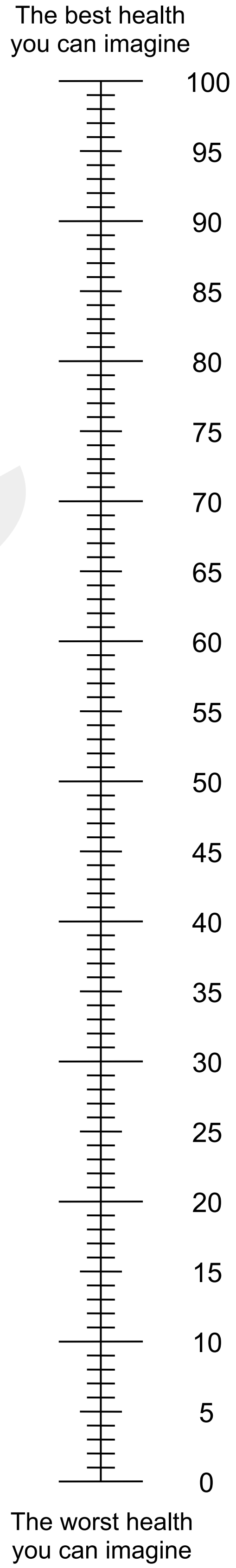

Sample

UK (English) © 2009 EuroQol Group EQ-5D™ is a trade mark of the EuroQol Group
